# A novel method of assessing balance and postural sway in patients with hypermobile Ehlers-Danlos syndrome

**DOI:** 10.3389/fmed.2023.1135473

**Published:** 2023-06-16

**Authors:** Miguel Whitmore, Brittany Barker, Katie Chudej, Ciarra Goines, Jenna Kester, Hannah Campbell, Anna Jeffcoat, Brynn Castleberry, Thomas William Lowder

**Affiliations:** Department of Exercise and Sport Science, University of Central Arkansas, Conway, AR, United States

**Keywords:** hEDS, balance, postural sway, center of gravity, proprioception

## Abstract

Patients with hypermobile Ehlers-Danlos syndrome (hEDS) frequently suffer from poor balance and proprioception and are at an increased risk for falls. Here we present a means of assessing a variety of balance and postural conditions in a fast and non-invasive manner. The equipment required is commercially available and requires limited personnel. Patients can be repeatedly tested to determine balance and postural differences as a result of disease progression and aging, or a reversal following balance/exercise interventions.

## 1. Introduction

Ehlers-Danlos syndromes (EDS) comprise a group of 14 subtypes of heritable connective tissues, the most prevalent being hypermobile (hEDS) (1). Patients suffer frequent joint dislocations/subluxations, kinesiophobia, skin fragility and hyperextensibility chronic pain/inflammation, postural orthostatic tachycardia syndrome as well as mast cell activation syndrome (2, 3). Despite its identity as a connective tissue disorder, hEDS has frequently been demonstrated to produce multisystemic symptom dysregulation that impact crucial bodily functions. Patients with hEDS often go years without a definitive diagnosis, often diagnosed with fibromyalgia or anxiety. Indeed, pain is a leading complaint and cause of anxiety among hEDS patients, as well as a leading cause of exercise and physical therapy avoidance (2). Other than pain management, few treatment options exist for hEDS patients. A recent paper demonstrated that EDS, once thought to be a rare disease, may be more prevalent than previously cited (4). While several articles advocate the use of physical therapy in EDS patients, few studies have used this to treat and improve quality-of-life (Q of L) in patients with EDS.

Bissolotti et al. examined sit-to-stand (STS) tasks in individuals with Parkinson’s disease (5). As is the case in individuals with Parkinson’s disease, patients with hEDS also present with abnormal posture, presumably resulting from poor spinal alignment. In both populations, flexion of the hips and knees may result in skeletal deformities that may in turn affect posture (due to spinal, hip, and/or knee misalignment) and thus balance. Joint hypermobility may be symptomatic or asymptomatic and can result from not only the disease, but also from muscular imbalance. Indeed, muscular imbalances, proprioception, and balance should be addressed in hEDS patients (6). These muscle weaknesses and imbalances may be a result of lax joints (connective tissue) that either prohibit patients from exercising or instill a level of kinesiophobia that prevents them from attempting to exercise, despite recommendations for patients to do so (2). Balance is an essential aspect of nearly all upright activities and provides the framework for numerous movements. Balance is paramount in preventing accidental injuries from falls, which due to our bipedal nature, becomes an increasingly difficult and complex task requiring multiple bodily systems to work in tandem (7). Chief among these systems is proprioception, the body’s ability to ascertain its location in space (8). This system comprises a variety of distinct sensory receptors, providing the central nervous system (CNS) information regarding various aspects of body position.

The primary proprioceptors involved are the muscle spindles, which encode muscle length and position of the joints (9), Golgi tendon organs that signal tension produced by the muscles (10), and vestibular sensors that relay the position of the head relative to the ground. Other sensory receptors within joints and skin, such as cutaneous afferents, have been shown to play a role in determining the body’s joint positions (11). Together, these afferent signals are integrated to produce an accurate internal model of the body state from which the CNS can devise effective motor responses. In balance, these signals are used to determine the body’s center of mass (CoM) (7).

Biomechanics classically defines CoM as the imaginary point at which a body’s mass acts around, while a “line” drawn from the CoM to the ground reveals the center of gravity (CoG) (12). Balance refers to the ability to maintain the CoM within the base of stability (that is, the feet and the area between them) while minimizing large displacements from its center. Different body systems will utilize this interaction in a variety of ways. For example, during quiet stance, the CoM is held over the center of the base, the goal being to minimize excursions from this point (13). If the CoM were centered perfectly, postural equilibrium would passively be maintained. This, however, is impossible due to physiological tremors, which produce oscillations in CoM position (14). Proprioceptors detect these passive movements and in turn shape postural muscle responses that realign CoM, highlighting the importance of accurate proprioception to maintain proper balance. Populations with impaired proprioception, such as individuals with hEDS, reflect this by demonstrating a worsened level of stability and a greater sway magnitude, resulting in an increased risk of sustaining a fall-induced injury.

A decreased level of proprioceptive acuity has long been associated with EDS, and deficits have been demonstrated within both the hand and knee joints in this population (15). These findings are believed to underpin alterations in balance during complex motor tasks, such as gait, concomitant with largely altered joint kinematics due to a defect in connective tissues, thus altering balance during passive standing, thus demonstrating a greater CoG displacement and a need for balance training in this population (16, 17). Despite these findings, no study has yet analyzed dynamic postural control within hEDS, which may serve as an indicator of not only functional stability, but also the pathophysiology underlying poor balance. Therefore, the purpose of the current study was to characterize the balance deficits associated with hEDS by providing an analysis of dynamic postural control. These findings may have implications for therapeutic treatments and/or interventions designed to improve postural stability in individuals with hEDS.

## 2. Materials and methods

This study was approved by our Institutional Review Board. All participants read and then signed the informed consent. Individuals with hEDS were assessed for inclusion based on a prior clinical diagnosis or after meeting all three criteria listed on the Diagnostic Criteria for hEDS checklist (18). Hypermobility was verified prior to balance testing using the Beighton criteria, which scores participants based on the number of hypermobile movements they display (19). Exclusion was based on the diagnostic criteria for hEDS checklist, and a Beighton score of ≤4 evaluated with the investigators prior to testing. A total of 56 patients were tested (*n* = 53 female, 2 male, 1 non-binary). The patient identifying as non-binary is a biological male.

We assessed each patient via a balance test to determine each patient’s mobility, fall risk, and ability to move unassisted by utilizing a widely used balance test for determining balance and stability (NeuroCom Balance Manager). This test is used in physical therapy clinical settings, and as most hEDS patients are self-described as “clumsy” with poor spatial awareness, this test is a validated and easily administered test for these measures. We will assess this using a validated balance test. While this was originally developed for individuals with traumatic brain injury, such as concussions, no balance test exists for hEDS patients. We hope to validate the use of the NeuroCom Balance Manager as a means of assessing balance, proprioception, and postural sway in hEDS patients.

Balance testing was performed using the NeuroCom VSR Sport force plate. The plate was positioned away from any extraneous objects with the computer monitor placed in front of the participant. Screen height was set at or slightly below eye level, allowing for visual aid during all tests. Five tests were selected based on their validity in analysis of postural stability and are briefly summarized below. Tests were performed in the order presented with breaks if required due to fatigue. Experimenters stood around the participants to eliminate falling.

### 2.1. Stability evaluation test

The stability evaluation test (SET) analyzes a participant’s functional balance control. Though the standard protocol is performed with the eyes closed, the test was performed with eyes open to better analyze the variety of stances taken in everyday activities, as well as due to the fact the patients with hEDS very often report proprioception and balance issues.

Stability was assessed in the double (DB), single (SG), and tandem (TD) standing positions. In the DB stance, participants placed both feet together and positioned them in the center of the force plate (Supplementary Figure 1). SG stance was performed on the indicated non-dominant foot which was similarly placed in the center of the plate (Supplementary Figure 2). Participants were instructed not to brace or use their lifted leg for support. TD consisted of both feet placed in a line with the non-dominant foot behind the dominant foot (Supplementary Figure 3). Both feet were positioned along the vertical axis of the force plate with the point of contact between the heel and toe in the center. For all stances, participants placed their hands on their hips but were allowed to move them if necessary to maintain balance.

Stances were assessed in two conditions, either standing directly on the force plate (firm) or on a foam pad (foam) placed on top of the force plate, the latter which created an unstable surface. Stances in the firm condition were completed in the order DB, SG, TD before proceeding to the foam, with stances performed in the same order. Each test lasted for 20 s during which center of gravity (COG) sway velocity (SV) was recorded (deg/s).

### 2.2. Modified CTSIB test

The Modified CTSIB (MCTSIB) test examines sensory function by modifying sensory feedback through various testing conditions. Feet were positioned hip-width apart with ankles aligned along the horizontal axis of the force plate and arms placed down at the sides. Stability was again assessed on both firm and foam surfaces with each condition subdivided into eyes opened (EO) and eyes closed (EC) segments. Three trials lasting 10 s each were completed for each condition during which CoG sway velocity (deg/s) was recorded. The average from all completed trials were used in the analysis.

### 2.3. Limits of stability test

Limits of stability (LOS) analyzes the extent to which participants can volitionally position CoG without losing balance, thus serving as an accurate indicator of dynamic postural control. Feet were placed in the same position as during the MCTSIB. On the screen was a central box encircled by eight target boxes numbered 1–8, with each target box spaced 45° apart. A colored icon was displayed on the screen serving as a real-time representation of the individual’s CoG. Participants were instructed to move the CoG icon about the screen by shifting their weight, however necessary, to the center of each successive box while keeping both feet flat on the force plate.

Prior to the test, participants aligned their CoG within the center box and were made aware of each target that they would be moving toward. Once participants indicated they were ready to begin, the experimenter began recording. An auditory cue signaled the participant to move toward the correct box. Weight was shifted to position the CoG as close to the indicated target as possible. Trials lasted 20 s each irrespective of whether the target box was reached. After the first trial was completed, the process was repeated for the remaining target boxes proceeding clockwise.

Five variables were recorded as output from the force plate: reaction time (RT), movement velocity (MV), endpoint excursion (EPE), maximum excursion (ME), and directional control (DLC). RT refers to the time in seconds between the auditory cue and onset of volitional movement. MV was the average velocity (deg/s) at which CoG was shifted toward the target. EPE was the maximum distance willingly traveled toward the target (%) in the first continuous movement from the center. ME is the greatest distance achieved in the direction of the target (%). Directional control (%) indicated the amount of movement toward the target box (%).

### 2.4. Unilateral stance test

The unilateral stance test (UST) evaluates postural stability of each leg individually. Feet were positioned according to the NeuroCom VSR protocol, standing with the foot off-center in its respective direction (i.e., left foot on the left side) with ankles aligned along the horizontal axis of the plate. During the test, participants were instructed not to brace themselves with the lifted leg. However, arm movements were allowed to maintain balance if necessary. Experimenters assisted participants with maintaining balance in the testing position until recording began. Balance was assessed on each foot with EO and EC. Three trials lasting 10 s each were completed for each condition before proceeding to the next (a total of six stances performed on each foot). CoG sway velocity (deg/s) was measured and the reported values for each condition are the average of all completed trials.

### 2.5. Rhythmic weight shift test

The rhythmic weight shift test (RWS) assesses dynamic postural control by analyzing the speed in which participants can move their CoG about their base of support. Stance and screen position were the same as described for the LOS test, with the CoG being displayed in the same manner. A second indicator icon was also displayed, serving as a visual cue for speed of weight shift, as it moved in a metronome-like fashion between two parallel lines placed on opposite sides of the screen. Upon reaching one of the lines, an auditory cue would sound, and the indicator’s movement would shift toward the opposite direction. Transit time of the indicator between lines decreased progressively with each speed condition, with 3 s for slow, 2 s for moderate, and 1 s for fast. Each condition was performed in this sequence first in the lateral (left to right) and then anterior to posterior (forward and backward) directions. Each participant performed one trial at each speed and in each direction (a total of six assessments).

In performing the test, participants were instructed to match CoG movement with that of the indicator as closely as possible. Recording began once a consistent pattern of movement emerged and was maintained for at least 3 s. Variables recorded were MV (deg/s) and DLC (%) in the respective direction of movement for each condition.

## 3. Results

**Figure 1:**
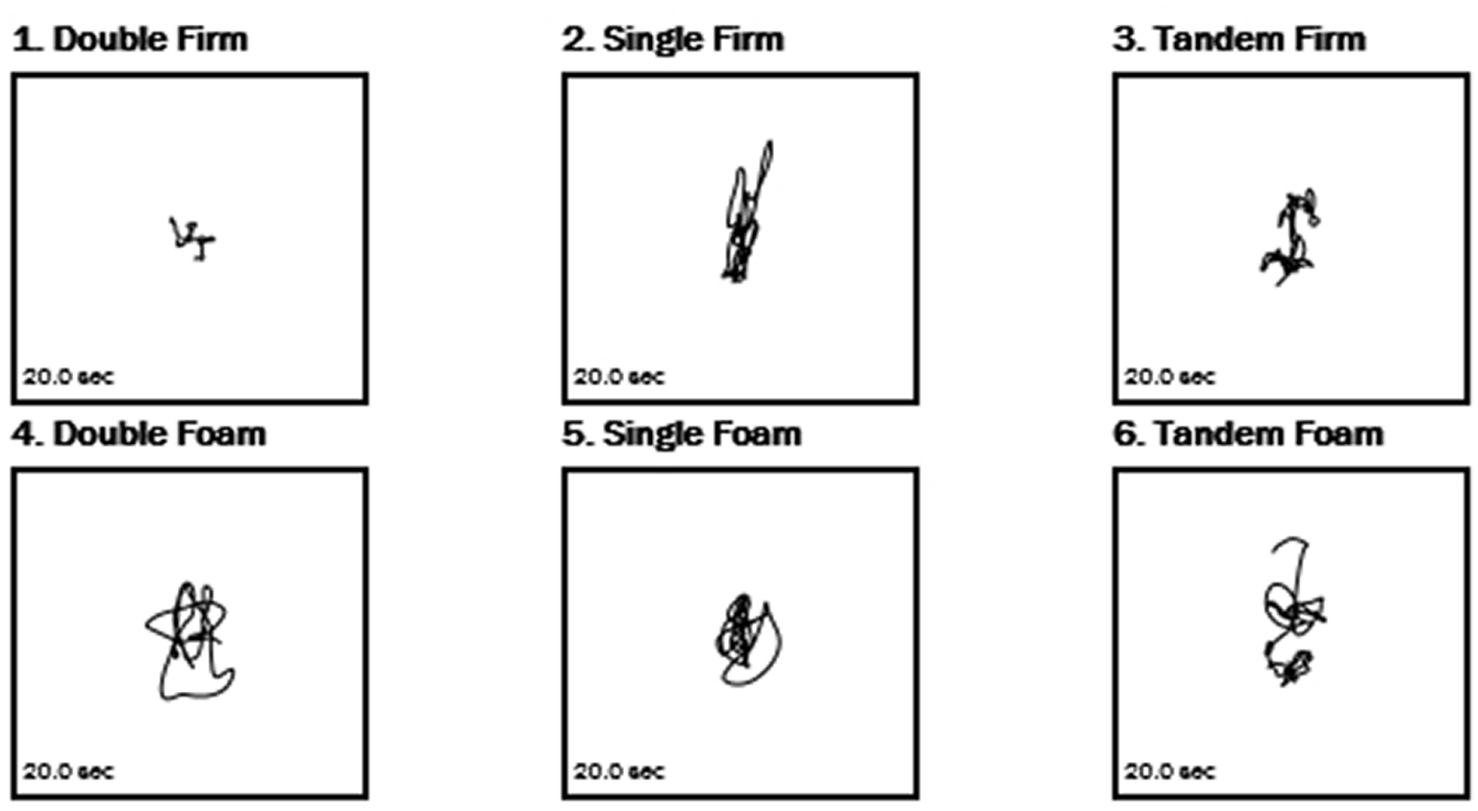
Sample center of gravity tracings from the stability evaluation test.

**Figure 2:**
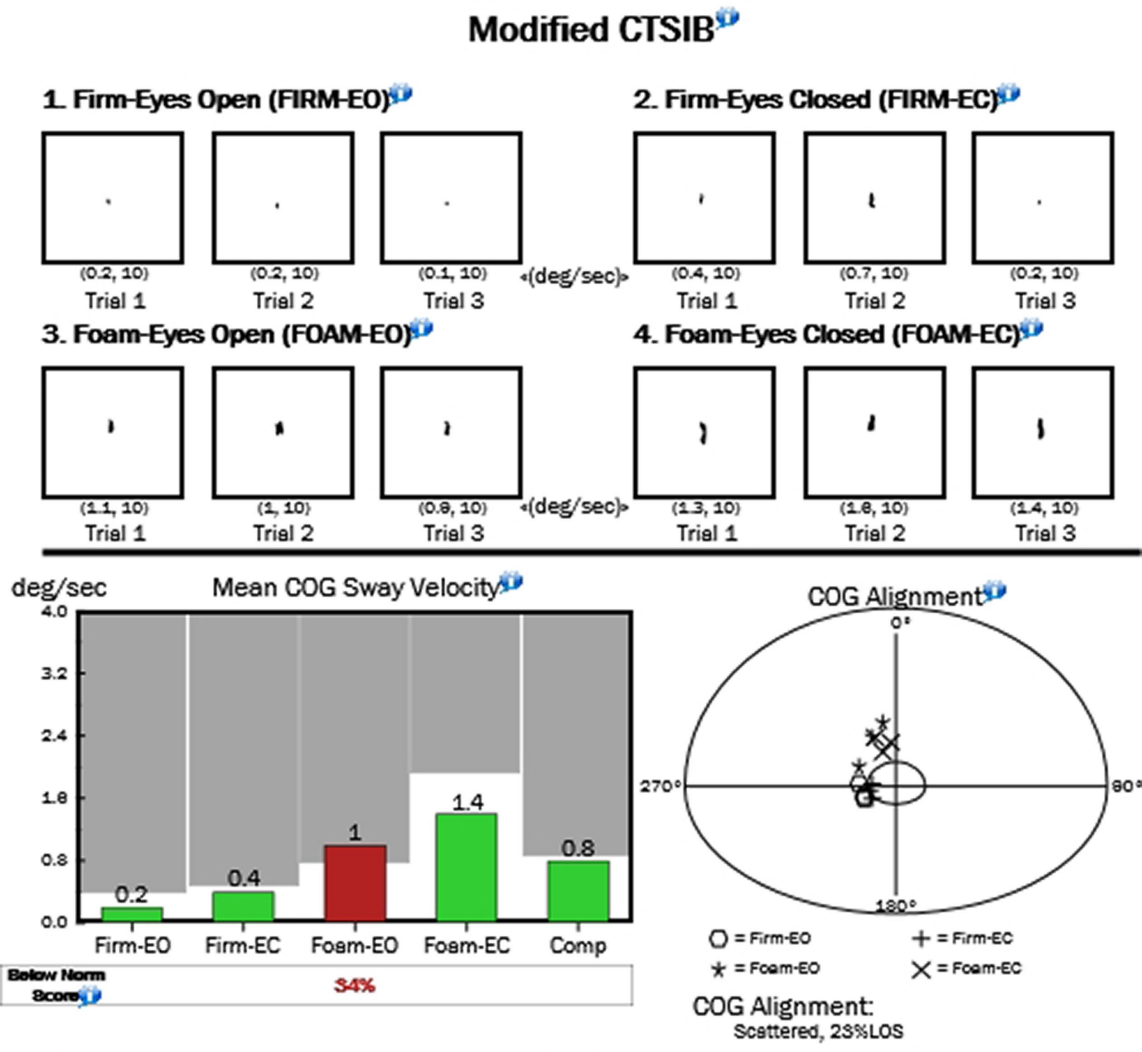
Sample center of gravity from the MCTSIB test.

**Figure 3:**
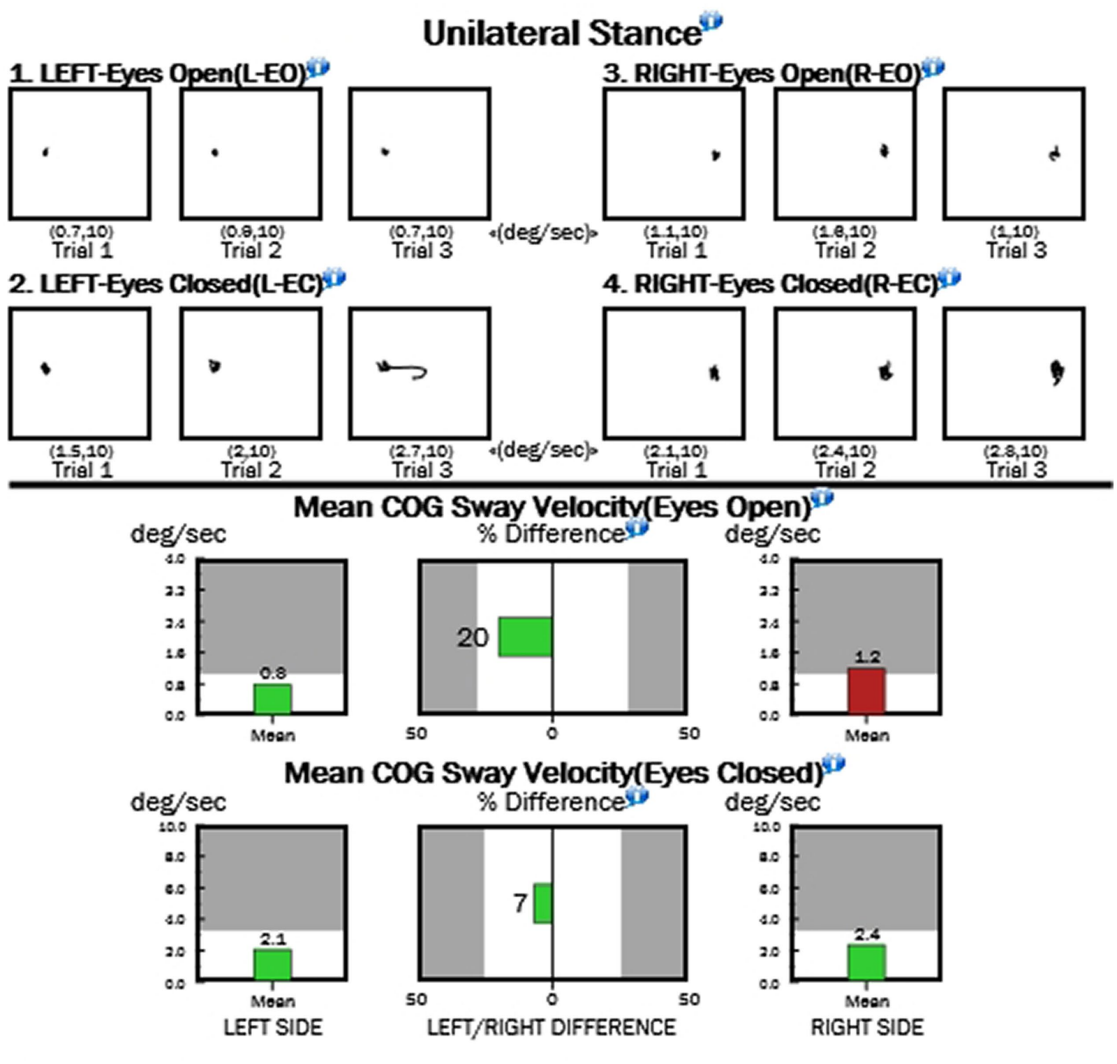
Sample results from the unilateral stance test.

While we do not have comparisons for non-hEDS populations, we present here a data set with 56 hEDS patients (*n* = 53 female, 2 male, 1 non-binary who is a biological male). Table 1 presents the results of the stability evaluation test (SET), while Figure 1 shows an image of the test with the center of gravity (COG) evident for each test. The flat surface (non-foam) was significantly more stable for all three conditions compared with the foam stability (DB, *p* > 0.0001; SG, *p* > 0.0001; and TD, *p* = 0.003). Means for each condition are shown in Table 1. For the MCTSIB, which compared firm vs. foam for both eyes open and eyes closed conditions, highly significant differences were seen for both conditions (*p* = 0.0005 and *p* > 0.0001 for firm EO vs. firm EC and foam EO vs. foam EC, respectively; Figure 2). Means for each condition are shown in Table 2. The unilateral stance test results are shown in Table 3. For mean center of gravity sway velocities, which examined sway while standing on one leg, significant differences (*p* > 0.0001) were observed when comparing standing on the left leg with eyes open vs. closed, as well as on the right leg with eyes open vs. closed (Figure 3).

**Table 1:** Results from the stability evaluation test.

Stability evaluation						
	DB	SG	TD	Foam DB	Foam SG	Foam TD
Mean	0.58	1.19	1.12	0.96	1.58	1.14
SEM	0.04	0.07	0.08	0.05	0.13	0.06
SD	0.26	0.51	0.58	0.35	0.96	0.45
Range	1.5	2.5	3.5	1.6	5.39	1.95
Minimum	0.2	0.5	0.3	0.4	0.6	0.48
Maximum	1.7	3.0	3.8	2	5.99	2.43

**Table 2:** Results from the modified CTSIB (MCTSIB) test.

Modified CTSIB (MCTSIB) test				
	Firm EO	Firm EC	Foam EO	Foam EC
Mean	0.43	0.58	0.76	1.37
SEM	0.03	0.05	0.06	0.08
SD	0.21	0.39	0.41	0.58
Range	0.9	2.1	2.2	2.7
Minimum	0.2	0.2	0.2	0.4
Maximum	1.1	2.3	2.4	3.1

**Table 3:** Results from the unilateral stance test.

Unilateral stance test (sway velocities in degrees per second)				
	Left EO	Right EO	Left EC	Right EC
Mean	1.1	1.16	3.94	3.47
SEM	0.09	0.10	0.47	0.42
SD	0.61	0.68	3.36	2.97
Range	4.1	4.0	11.1	11.0
Minimum	0.5	0.6	0.9	1.0
Maximum	4.6	4.6	12	12

**Figure 4:**
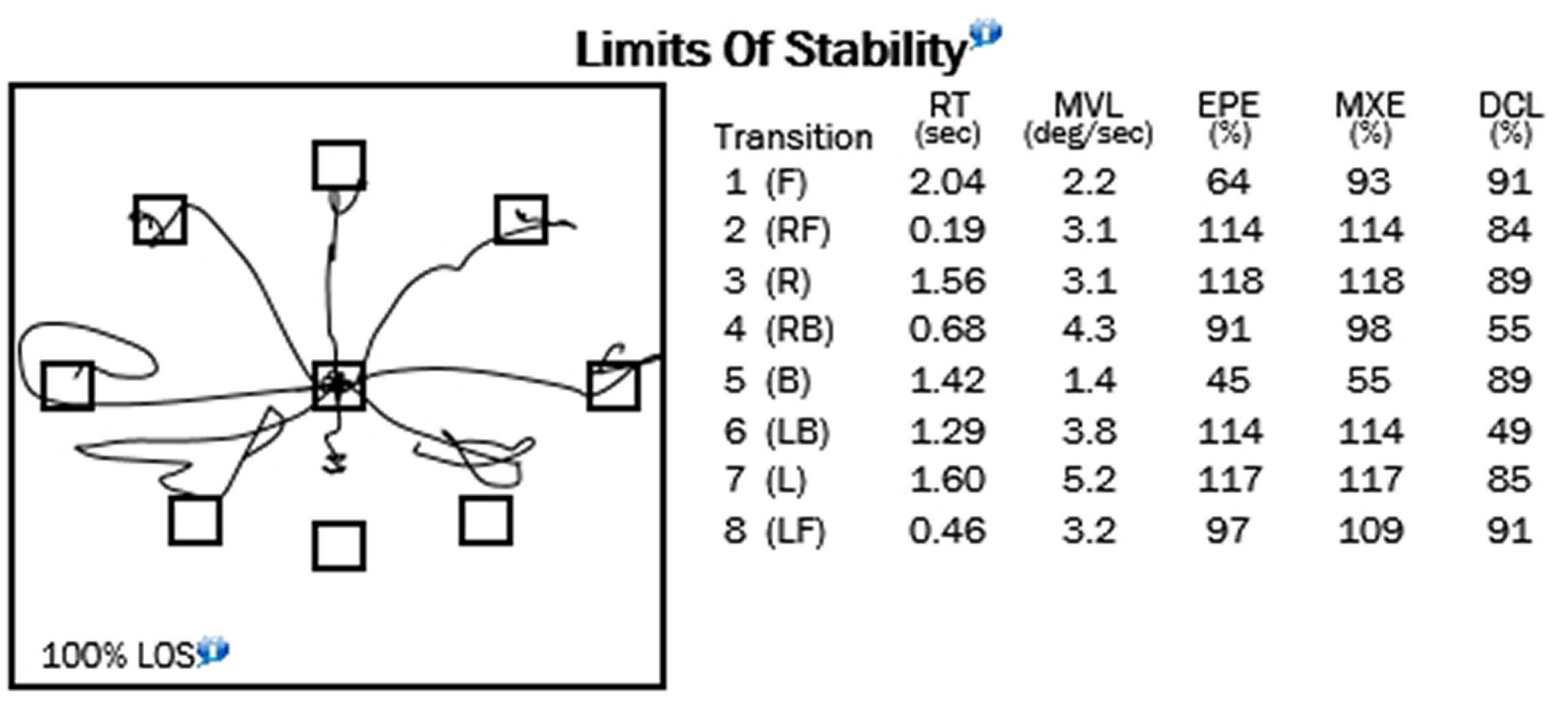
Example of limits of stability test.

**Figure 5:**
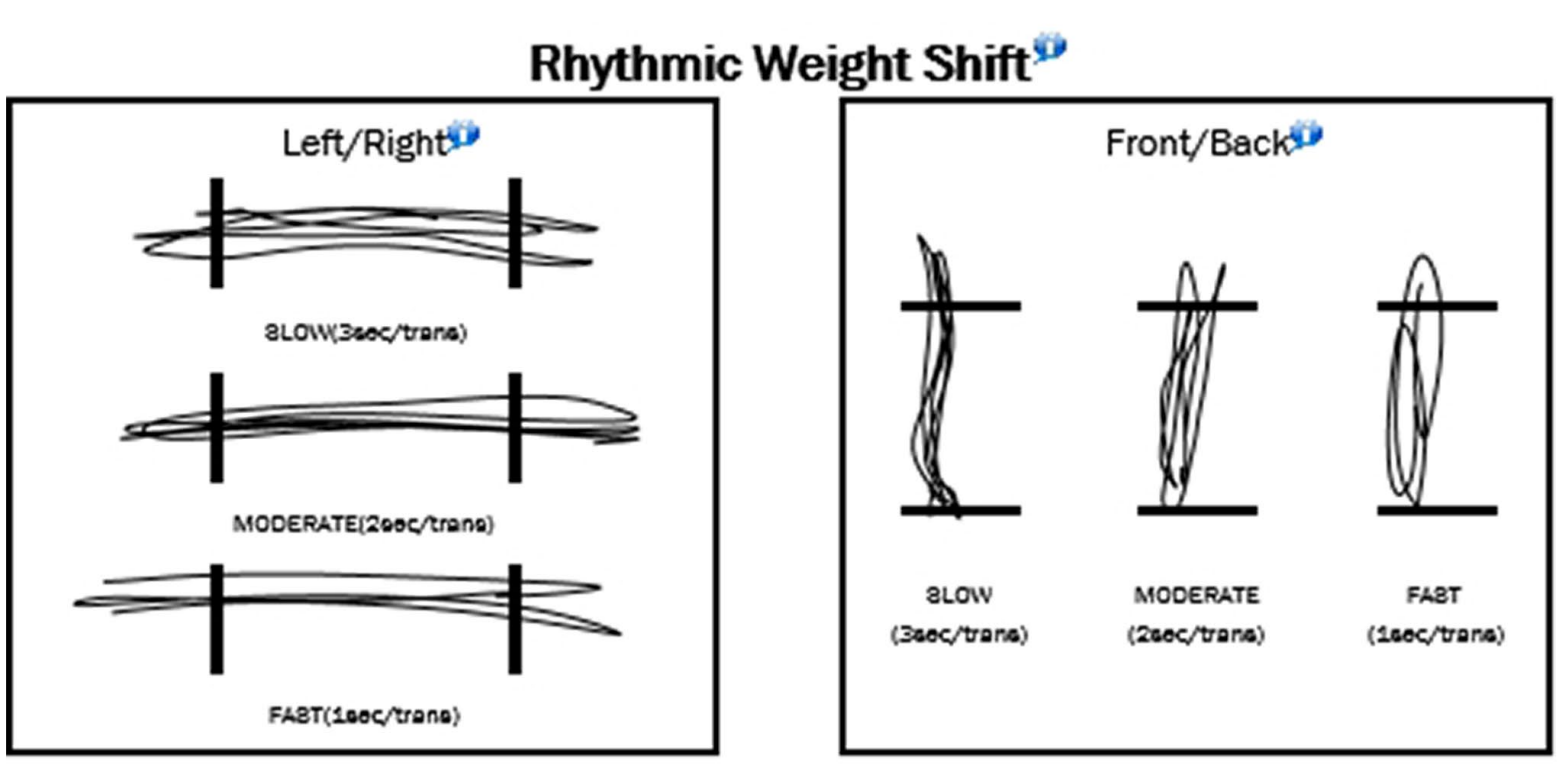
Example of the rhythmic weight shift tests.

Tables 4 show the means for limits of stability (reaction time; movement velocity in degrees per second; and percentages of endpoint excursion, maximum excursion, and directional control; Figure 4). Table 5 shows the results of the Rhythmic Weight Shirt for all directions (left to right and front to back velocities, in degrees per second; and directional control right to left and front to back as a percentage; Figure 5).

**Table 4:** Limits of stability tests.

Limits of stability test results				
	Forward	Back	Right	Left
**Reaction time**				
Mean	1.05	0.82	0.97	0.91
SEM	0.06	0.04	0.06	0.04
SD	0.4	0.28	0.42	0.31
Range	1.88	1.16	2.13	1.5
Minimum	0.35	0.24	0.24	0.3
Maximum	2.23	1.4	2.37	1.8
**Movement velocity (degrees per second)**				
Mean	3.35	2.29	4.24	12.09
SEM	0.15	0.13	0.28	7.54
SD	1.13	0.95	2.01	54.91
Range	4.7	4.6	9.5	402.6
Minimum	1.2	0.5	1.0	1.4
Maximum	5.9	5.1	10.5	404
**Endpoint excursion (percentage)**				
Mean	67.06	49.74	79.85	91.92
SEM	2.46	2.41	2.91	2.67
SD	17.94	17.53	21.16	19.44
Range	75.0	91.0	94.0	88.0
Minimum	27.0	6.0	27.0	35.0
Maximum	102.0	97.0	121.0	123.0
**Maximal excursion (percentage)**				
Mean	88.08	67.66	104.21	108.32
SEM	1.93	2.55	2.04	1.85
SD	14.07	18.58	14.88	13.44
Range	74.0	97.0	81.0	64.0
Minimum	38.0	6.0	44.0	67.0
Maximum	112.0	103.0	125.0	131.0
**Directional control (percentage)**				
Mean	84.62	68.19	80.72	80.89
SEM	0.97	1.83	1.0	0.83
SD	7.04	13.2	7.29	6.06
Range	37.0	53.0	30.0	29.0
Minimum	59.0	35.0	62.0	60.0
Maximum	96.0	88.0	92.0	89.0

**Table 5:** Results from rhythmic weight shift tests.

Rhythmic weight shift			
	Slow	Medium	Fast
**Left/right velocity (degrees per second)**			
Mean	3.48	5.26	11.17
SEM	0.05	0.1	0.32
SD	0.4	0.72	2.34
Range	1.5	3.5	11.0
Minimum	2.8	3.2	5.1
Maximum	4.3	6.7	16.1
**Directional control right to left (percentage)**			
Mean	88.7	87.24	89.83
SEM	0.49	0.51	0.61
SD	3.59	3.72	4.42
Range	18.0	20.0	27.0
Minimum	73.0	73.0	68.0
Maximum	91.0	93.0	95.0
**Front/back velocity (degrees per second)**			
Mean	2.29	3.29	5.55
SEM	0.06	0.06	0.2
SD	0.43	0.61	1.48
Range	2.0	2.9	6.4
Minimum	1.4	1.5	2.3
Maximum	3.4	4.4	8.7
**Directional control front to back (percentage)**			
Mean	73.67	76.11	76.36
SEM	1.84	2.42	1.91
SD	13.49	17.61	13.94
Range	70.0	90.0	71.0
Minimum	21.0	1.0	19.0
Maximum	91.0	93.0	90.0

A control group (*n* = 10, 8 female and 2 male) were tested to compare hEDS patients with non-hEDS patients. None of the control subjects reported any known neurological disease, head injury, hypermobility, or balance issues. Tables 6–10 show control subject data.

**Table 6:** Results from the stability evaluation test (control non-hEDS).

Stability evaluation						
	DB	SG	TD	Foam DB	Foam SG	Foam TD
Mean	0.55	0.91	0.72	0.83	0.87	0.87
SEM	0.03	0.04	0.04	0.06	0.05	0.07
SD	0.09	0.14	0.12	0.20	0.17	0.22
Range	0.30	0.40	0.40	0.50	0.40	0.70
Minimum	0.40	0.70	0.60	0.60	0.70	0.60
Maximum	0.70	1.10	1.00	1.10	1.10	1.30

**Table 7:** Results from the modified CTSIB (MCTSIB) test (control non-hEDS).

Modified CTSIB (MCTSIB)				
	Firm EO	Firm EC	Foam ED	Foam EC
Mean	0.36	0.32	0.51	0.77
SEM	0.07	0.25	0.03	0.06
SD	0.23	0.79	0.10	0.18
Range	0.80	0.20	0.30	0.50
Minimum	0.20	0.20	0.40	0.60
Maximum	1.00	0.40	0.70	1.10

**Table 8:** Results from the unilateral stance test (control non-hEDS).

Unilateral stance test (sway velocities in degrees per second)				
	Left EO	Right EO	Left EC	Right EC
Mean	0.75	0.72	1.56	1.51
SEM	0.05	0.04	0.09	0.08
SD	0.14	0.14	0.30	0.24
Range	0.50	0.50	1.10	0.90
Minimum	0.50	0.50	1.00	1.00
Maximum	1.00	1.00	2.00	1.90

**Table 9:** Limits of stability (control non-hEDS).

Limits of stability test results				
	Forward	Back	Right	Left
**Reaction time**				
Mean	0.77	0.58	0.60	0.64
SEM	0.10	0.02	0.05	0.05
SD	0.32	0.16	0.06	0.17
Range	1.02	0.58	0.46	0.51
Minimum	0.48	0.37	0.42	0.52
Maximum	1.50	0.95	0.88	1.03
**Movement velocity (degrees per second)**				
Mean	5.21	3.51	6.56	7.42
SEM	0.59	0.42	1.25	0.75
SD	1.87	1.33	3.96	2.37
Range	5.2	4.60	13.8	7.90
Minimum	2.60	1.60	2.8	2.90
Maximum	7.80	6.20	16.6	10.8
**Endpoint excursion (percentage)**				
Mean	77.4	63.5	98.9	107.2
SEM	4.98	5.15	4.79	5.82
SD	15.7	16.3	15.2	18.4
Range	50	57	45	55
Minimum	50	43	79	82
Maximum	100	100	124	137
**Maximal excursion (percentage)**				
Mean	88.5	81.1	115	113.8
SEM	2.64	4.30	2.86	4.84
SD	8.34	13.6	9.04	15.3
Range	20	38.0	32.0	49.0
Minimum	80	62.0	103	88.0
Maximum	100	100	135	137
**Directional control (percentage)**				
Mean	84.1	73.5	81.4	80.7
SEM	1.55	2.65	1.35	1.52
SD	4.91	8.38	4.27	4.81
Range	15.0	23.0	12.0	15.0
Minimum	75.0	62.0	75.0	72.0
Maximum	90.0	85.0	87.0	87.0

**Table 10:** Results from rhythmic weight shift tests (control non-hEDS).

Rhythmic weight shift			
	Slow	Medium	Fast
**Left/right velocity (degrees per second)**			
Mean	3.48	5.26	11.17
SEM	0.05	0.10	0.32
SD	0.40	0.72	2.34
Range	1.50	3.50	1.10
Minimum	2.80	3.20	5.10
Maximum	4.30	6.70	16.1
**Directional control right to left (percentage)**			
Mean	83.87	87.24	89.8
SEM	0.49	0.51	0.61
SD	3.59	3.72	4.42
Range	18.0	20.0	27.0
Minimum	73.0	73.0	68.0
Maximum	91.0	93.0	95.0
**Front/back velocity (degrees per second)**			
Mean	2.29	3.29	5.55
SEM	0.06	0.08	0.20
SD	0.43	0.61	1.48
Range	2.0	2.90	6.40
Minimum	1.40	1.50	2.30
Maximum	3.40	4.40	8.70
**Directional control front to back (percentage)**			
Mean	73.7	78.1	76.4
SEM	1.84	2.42	2.50
SD	13.5	17.6	18.2
Range	70.0	90.0	93.0
Minimum	21.0	1.0	0
Maximum	91.0	91.0	93.0

## 4. Statistical analysis

Data were tabulated for each of the variables (MCTSIB, LOS, UST, RWS) for each of the tests assessed (e.g., reaction time, movement velocity, endpoint excursion, maximal excursion, and directional control for LOS) and the mean (±SEM and SD), range, minimum, and maximum values were determined.

## 5. Discussion

Here, we provide normative data regarding postural sway and dynamic stability in hEDS for the purpose of better characterizing balance within this population. As patients experience more frequent falls, increasing their risk of dislocations/subluxations, a better understanding of balance is essential in designing effective exercise-based interventions to improve QoL. Precluding general guidelines, however, are the wide range of symptoms and comorbidities associated with hEDS which may contribute toward reducing balance. Decreased proprioception (15), as well as muscle weakness and hypotonia (20), make the effective coordination of motor responses difficult, causing patients to be more unstable during upright tasks. These results may serve as a baseline for future testing of hEDS patients, particularly those with proprioception and balance issues. We are presently investigating the results of an exercise intervention on repeated measures of these tests.

Hyper-elasticity of soft tissue reduces the ability of tendons and ligaments to mediate force transmission and provide structural support, respectively, (3), resulting in greater postural instability. Additionally, the presence of postural orthostatic tachycardia syndrome, a prevalent comorbidity in hEDS (21), can impair balance as episodes produce immediate and/or prolonged feelings of dizziness or nausea. Patients experience these symptoms, among others, to highly varying degrees (22), making any broad explanations for poor balance difficult; thorough assessments of individual balance are essential for identifying specific areas in which training is needed (23). Our presented data will aid in assessing and improving balance in patients with hEDS by providing a benchmark for comparison in several aspects of functional stability.

Patients with EDS often suffer from POTS (postural orthostatic tachycardia syndrome) which can affect their balance. Many patients suffer from dislocations or subluxations following these falls. One goal of our ongoing study is to reduce falls by strengthening skeletal muscle and improving coordination, thus reducing falls. We did not perform so-called “rocker” balance tests, which require a great deal of skill to perform, due to the complexity of the movement. Our interest lies in the general, overall balance of each patient. This test is a non-invasive measure, and each patient had a minimum of three investigators present during each test to ensure safety.

Although inadequate alone, intrinsic stiffness of connective tissue plays a contributory role in maintaining balance due to the provided structural support and force transmission (24). Altered structural properties are believed to broadly impact soft tissue in hEDS which may contribute toward loss of balance. Elasticity of both ligaments and tendons have been shown to increase the magnitude of postural sway when structurally abnormal. Mechanical ankle instability has been shown to produce greater variance in postural sway (25); a comparison between those with mechanical and functional instability found that only those with mechanical ankle instability experienced greater postural sway than controls (26), indicating that connective tissue mechanics may impair quiet stance more than neuromuscular deficits. Similar findings have been reported in analyses of Achilles tendon, which have intrinsic properties that contribute to ankle joint stiffness during quiet stance (27). Greater magnitudes of postural sway can be inversely correlated with tendon stiffness in average populations (28) and during physiologically induced changes in tissue elasticity (29), further indicating the relative importance of tissue mechanical properties in maintaining balance. Hyper-elastic properties of both ligaments and tendon structures are well documented in hEDS (3), indicating that structural deficits may be a secondary malefactor in postural instability. Measurements of postural sway are therefore important in this population as greater variability in postural sway may reflect individuals’ degree of expressed hypermobility (22).

## 6. Future directions and clinical implications

We are at present investigating if an individualized exercise training intervention, consisting of balance and strength training using a limited range of motion, will have an impact on these measures. A recent paper demonstrated that balance can be significantly improved in frail, older individuals recovering from COVID-19 after intensive care. To date we have only re-tested one individual, but we did see improvements in their overall balance. Few studies examining the benefits of exercise in those with hEDS exist. To our knowledge this is the only study that has specifically examined multiple facets of balance and stability in this patient population. The data presented herein may provide a means for clinicians to assess progress in balance and posture stability in those with hEDS or other connective tissue disorders.

## Data availability statement

The raw data supporting the conclusions of this article will be made available by the authors, without undue reservation.

## Ethics statement

The studies involving human participants were reviewed and approved by Institutional Review Board at the University of Central Arkansas. The patients/participants provided their written informed consent to participate in this study.

## Author contributions

BB, KC, CG, JK, HC, AJ helped with testing patients. MW and TL tested patients, wrote/edited the manuscript, and analyzed the data. All authors contributed to the article and approved the submitted version.

## Funding

This study was funded in part by a grant from Arkansas IDeA Network of Biomedical Research Excellence (INBRE) Summer Manuscript Support (SMS) from the University of Arkansas Medical Sciences and the University of Central Arkansas University Research Council.

## Conflict of interest

The authors declare that the research was conducted in the absence of any commercial or financial relationships that could be construed as a potential conflict of interest.

## Publisher’s note

All claims expressed in this article are solely those of the authors and do not necessarily represent those of their affiliated organizations, or those of the publisher, the editors and the reviewers. Any product that may be evaluated in this article, or claim that may be made by its manufacturer, is not guaranteed or endorsed by the publisher.
