# Supplementary figures and images for: A novel method of assessing balance and postural sway in patients with hypermobile Ehlers-Danlos syndrome
Source: Front Med (Lausanne). 2023 Jun 16;10:1135473. doi: 10.3389/fmed.2023.1135473 (PMC10312239; doi:10.3389/fmed.2023.1135473)

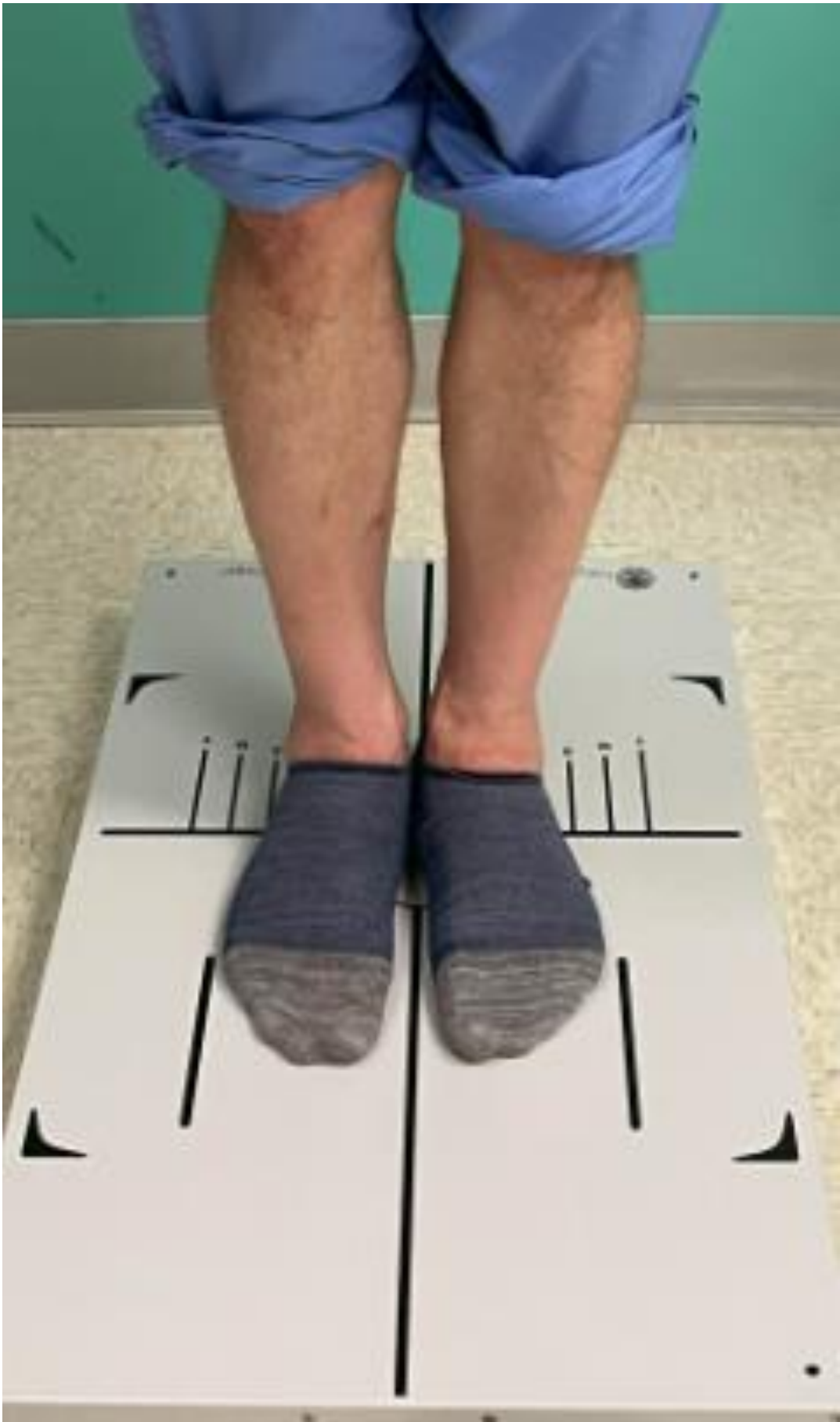

Image 1. DB stance

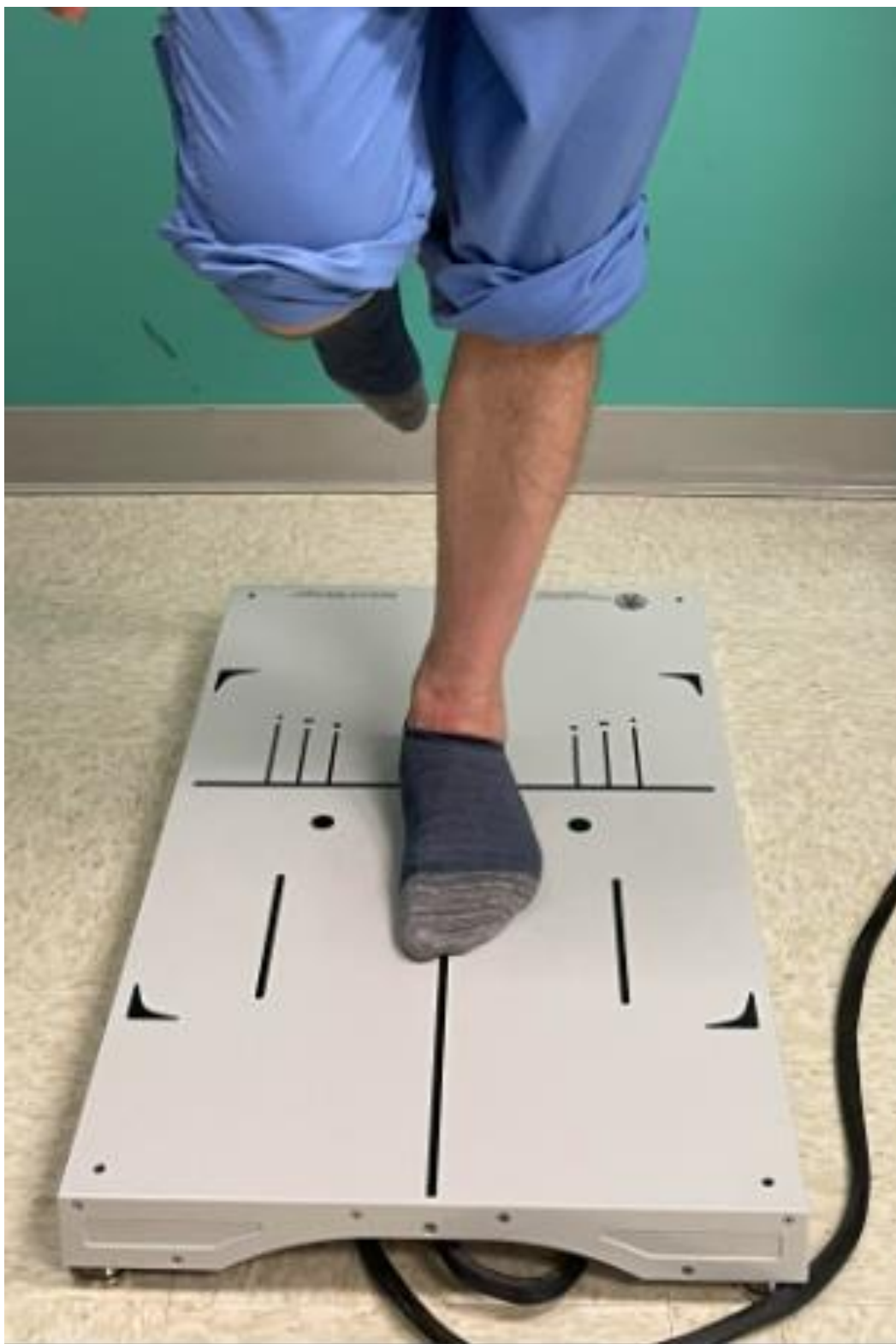

Image 2. SG stance.

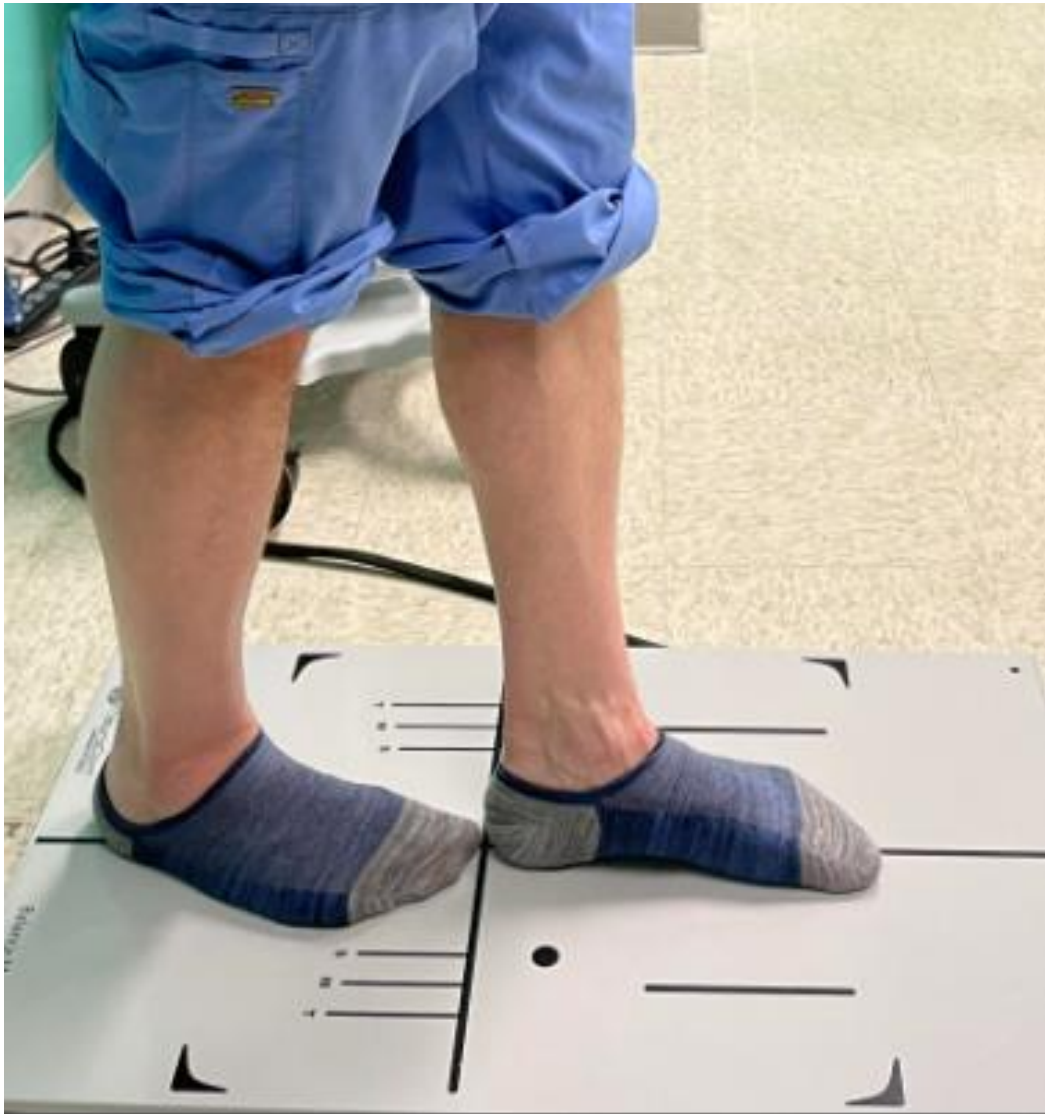

Image 3. TD stance.

Supplement: Supplementary file 1 [file Data_Sheet_1.PDF]
